# GLEANER: a web server for GermLine cycle Expression ANalysis and Epigenetic Roadmap visualization

**DOI:** 10.1186/s12859-021-04217-1

**Published:** 2021-05-31

**Authors:** Shiyang Zeng, Yuwei Hua, Yong Zhang, Guifen Liu, Chengchen Zhao

**Affiliations:** Institute for Regenerative Medicine, Shanghai East Hospital, Shanghai Key Laboratory of Signaling and Disease Research, Frontier Science Center for Stem Cell Research, School of Life Science and Technology, Tongji University, Shanghai, 200092 China

**Keywords:** Bioinformatics, Epigenetics, Germline cycle

## Abstract

**Background:**

Germline cells are important carriers of genetic and epigenetic information transmitted across generations in mammals. During the mammalian germline cell development cycle (i.e., the germline cycle), cell potency changes cyclically, accompanied by dynamic transcriptional changes and epigenetic reprogramming. Recently, to understand these dynamic and regulatory mechanisms, multiomic analyses, including transcriptomic and epigenomic analyses of DNA methylation, chromatin accessibility and histone modifications of germline cells, have been performed for different stages in human and mouse germline cycles. However, the long time span of the germline cycle and material scarcity of germline cells have largely limited the understanding of these dynamic characteristic changes. A tool that integrates the existing multiomics data and visualizes the overall continuous dynamic trends in the germline cycle can partially overcome such limitations.

**Results:**

Here, we present GLEANER, a web server for GermLine cycle Expression ANalysis and Epigenetics Roadmap visualization. GLEANER provides a comprehensive collection of the transcriptome, DNA methylome, chromatin accessibility, and H3K4me3, H3K27me3, and H3K9me3 histone modification characteristics in human and mouse germline cycles. For each input gene, GLEANER shows the integrative analysis results of its transcriptional and epigenetic features, the genes with correlated transcriptional changes, and the overall continuous dynamic trends in the germline cycle. We further used two case studies to demonstrate the detailed functionality of GLEANER and highlighted that it can provide valuable clues to the epigenetic regulation mechanisms in the genetic and epigenetic information transmitted during the germline cycle.

**Conclusions:**

To the best of our knowledge, GLEANER is the first web server dedicated to the analysis and visualization of multiomics data related to the mammalian germline cycle. GLEANER is freely available at http://compbio-zhanglab.org/GLEANER.

**Supplementary Information:**

The online version contains supplementary material available at 10.1186/s12859-021-04217-1.

## Background

In mammals, genetic and epigenetic information is transmitted across generations through the germline cell development cycle (i.e., germline cycle) [1]. The germline cycle runs through the whole process of ontogenesis and contains three phases: preimplantation embryogenesis, primordial germ cell (PGC) development in postimplantation embryos and gametogenesis in individuals after birth [2]. During these three phases of the germline cycle, the cell potency of germline cells changes cyclically, accompanied by dynamic transcriptional changes and epigenetic reprogramming. A systematic study of these cyclical changes in the mammalian germline cycle will help us to understand the mechanism of genetic and epigenetic information transmission across generations.

In the past decade, studies that characterized the dynamics of transcriptional and epigenetic features reported the local relationships of these dynamics at different phases of the mammalian germline cycle by using high-throughput sequencing technologies. For example, in the preimplantation embryogenesis phase of the mouse germline cycle, Liu et al. reported that transcriptional activation is related to increasing H3K4me3 distribution in gene promoter regions [3]. In the PGC development phase of the mouse germline cycle, Lesch et al. reported that the transcription activated epigenetic marker H3K4me3 was established and that the repressive epigenetic marker H3K27me3 was removed in the active gene promoters [4]. Recent studies further proved the continuity of the germline cycle by linking adjacent phases; for example, it has been reported that global epigenetic reprogramming of the genome occurs during the preimplantation embryogenesis phase and early PGC development phase in the mammalian germline cycle [5, 6]. Both of these waves of epigenetic reprogramming result in a decrease in global DNA methylation along with epigenetic modification re-establishment to regulate transcription [7–9]. However, these findings are still difficult to extend to the whole germline cycle to explain the inheritance and transmission of genetic information across generations due to the following two limitations. First, owing to the large time span of the germline cycle, previous studies profiled only the transcriptome and epigenome in one or two phases of the germline cycle and lacked an understanding of gene expression dynamics and epigenetic regulation during the whole germline cycle. Second, several important and rare cell types in the mammalian germline cycle (especially migrating PGCs in the early gestational stages in humans and mice and prospermatogonia and mitotic oocytes in the late gestational stage in humans) are difficult to collect and characterize, so transcriptome and epigenome profiles are usually incomplete, leading to a deficiency in continuous dynamic trends of the transcriptome and epigenome during the whole germline cycle. Taking advantage of the continuity and integrality of the germline cycle, it is feasible to overcome these two limitations by integrating the existing transcriptional and epigenetic high-throughput sequencing data in the germline cycle and building an online web server that provides multiomics features, the genes with correlated changes in transcription and the overall continuous dynamic trends of the transcriptome and epigenome for an inputted gene. However, to the best of our knowledge, no such web server is currently available.

Here, we present GLEANER, a web server for human and mouse germline cycle expression analysis and epigenetic roadmap visualization, which integrates and provides visualization of transcriptional and epigenetic features in the germline cycle. GLEANER collected 1,764 RNA sequencing (RNA-seq) samples, 600 bisulfite sequencing (BS-seq) samples characterized for DNA methylation, 25 DNase I hypersensitive sites sequencing (DNase-seq) samples and 61 ATAC-seq samples characterized for chromatin accessibility, and 151 ChIP sequencing (ChIP-seq) samples and 20 Cleavage Under Targets & Release Using Nuclease (CUT&RUN) samples characterized for histone modification (80 H3K4me3 samples, 71 H3K27me3 samples, and 20 H3K9me3 samples) in the three phases of the human and mouse germline cycle. GLEANER takes a gene or a genomic region as input and reports the following outputs: (1) integrated analysis of the transcriptional and epigenetic dynamics of the input element in the germline cycle; (2) the genes whose transcriptional changes are most positively or negatively correlated with those of the input element, as well as the integrated data matrix download; (3) overall continuous change trends of transcriptional and epigenetic features at the different stages of germline cycle, including the uncharacterized intermediate cell stages. In summary, GLEANER unifies the transcriptional and epigenetic feature visualization of three phases of the germline cycle and provides similarity analysis and regression analysis, which can yield valuable clues for understanding the transmission mechanisms and regulatory functions of epigenetic modification in the mammalian germline cycle.

## Construction and content

### Data collection and preprocessing

**Fig. 1:**
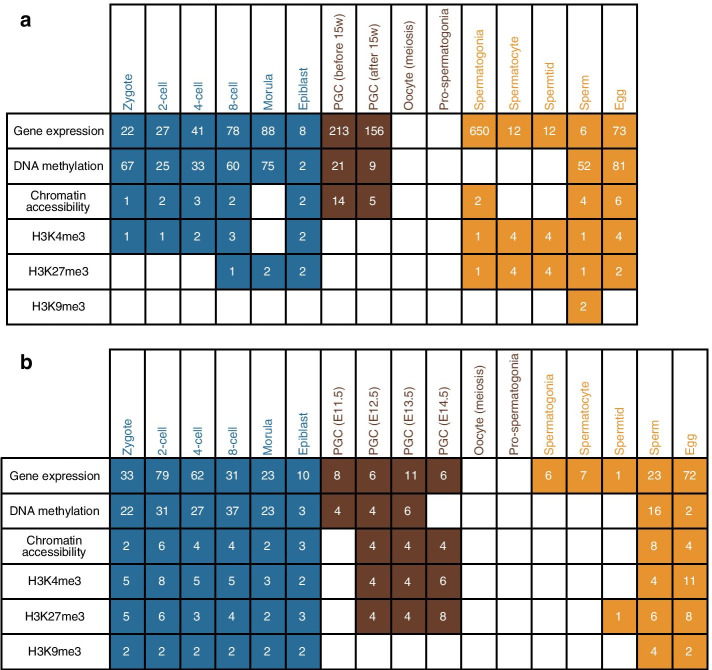
Summary of multiomics data in the human and mouse germline cycle collected in GLEANER. Number of multiomics data samples in the human (**A**) and mouse (**B**) germline cycle collected in GLEANER. Each row represents the type of multiomics data, including gene expression, DNA methylation, chromatin accessibility, and histone modification characteristics H3K4me3, H3K27me3, and H3K9me3. Each column represents the developmental stage of the germline cycle, which contains three major phases: preimplantation embryogenesis (blue), PGC development of postimplantation embryos (brown), and gametogenesis of after-birth individuals (orange). The numbers of table cells represent the sample number of each data type at each stage in the germline cycle

The high-throughput sequencing datasets characterized typical omics (i.e., transcriptomics, epigenomics of DNA methylation, chromatin accessibility, and three types of histone modifications) in mouse and human germline cycle are collected from a public database (Gene Expression Omnibus, GEO) [10, 11] (Fig. 1). To represent and investigate the transcriptional regulatory effects of epigenetic modifications, we focused on the gene promoter regions, defined as those regions − 2000 base pairs (bp) and + 500 bp around the transcription start sites. For these datasets with preprocessed data available, the preprocessed expression levels or the signals of epigenetic features were adopted. For those datasets that had only raw data available, raw reads were first trimmed using TrimGalore and then mapped to the reference genome (mm9 for mouse and hg19 for human). For RNA-seq data, the sequenced reads were mapped to the reference genome using TopHat (v2.1.1) [12] with default parameters. To make the expressions comparable between different samples, expression levels were measured as TPM values. For BS-seq data, BSMAP [13] was used to map the sequenced reads to the reference genome with parameters “-n 1 -r 0 -s 16” for WGBS data and parameters “-n 1 -r 0 -s 12 -D C-CGG” for RRBS data. The DNA methylation levels were quantified by using the mcall function in MOABS [14]. For ChIP-seq and CUT&RUN data, sequenced reads were mapped to reference genome using bowtie2 [15] with default parameter. The signals of epigenetic features were calculated from the piled-up reads using MACS [16] with parameters “–SPMR”, which generate profiling signals normalized to 1 million reads. For DNase-seq data and ATAC-seq data, bowtie2 was used to map the sequenced reads to the reference genome with default parameters. MACS pileup function was used to calculate the chromatin openness signal by using filtered fragments (less than 120 bp) with “–extsize = 50 –SPMR” to normalize signals to 1 million reads background. Transcriptional and epigenetic features from biological replicates were averaged for subsequent analysis. GLEANER also provides a visualized and downloadable page of these collected data including a data summary, the public accession numbers, and the related publication information.

### Data normalization

Samples from different researches may have batch effects. For different types of data, we used different strategies to eliminate the effects of different data sources or experimental batches. For the chromatin accessibility data and histone modification data, signal profiles were normalized per one million reads and calculated on genomic regions by using parameters “–SPMR” in MACS to control the sequencing depth to obtain relatively comparable values among different cell types. For DNA methylation data from BS-seq, due to the quantitative methylation levels calculation above, no more extra normalization processes were performed.

### Correlated changing genes selection

To select the genes with correlated transcriptional changes, we calculated the Pearson correlation coefficient (PCC) and the correlation test *p*-value between genes based on the expression levels in the germline cycle. The correlation calculations were performed using the R package Hmisc. Genes with a *p*-value < 0.01 and a PCC > 0.8 were selected as the positively correlated candidates, and genes with a *p*-value < 0.01 and a PCC <  − 0.8 were selected as the negatively correlated candidates.

### Regression model construction and overall continuous dynamic trend visualization

For each gene, different regression models, which included linear model, logarithmic model, and polynomial (quadratic, cubic, and quartic) model, were constructed and implemented by the ECharts Statistics module using the gene expression level or the average epigenetic signals within the gene promoter. With these regression models and their specific parameters, the overall continuous dynamic trends of the input transcriptional level or epigenetic features among the developmental stages in the mammalian germline cycle were demonstrated. The mean square errors of the predictions were calculated and displayed as a validity estimation measurement.

### Webserver implementation

In this study, the LAMP (i.e., Linux operating system, Apache web server, MySQL database, and PHP programming language) architecture was used to build an online platform (Additional file 1: Fig. 1). Through the data preprocessing above, we obtained normalized transcriptional and epigenetic features in the mouse and human germline cycles. A MySQL database was built as the back end of GLEANER. Programmed PHP scripts were used to construct the front end of the web page and connect with the back-end database. Subsequently, we used the open-source web framework Bootstrap for interactive interface establishment. The ECharts [17] library in JavaScript was used for statistical calculation and feature visualization in GLEANER.

### Utility and discussion

**Fig. 2:**
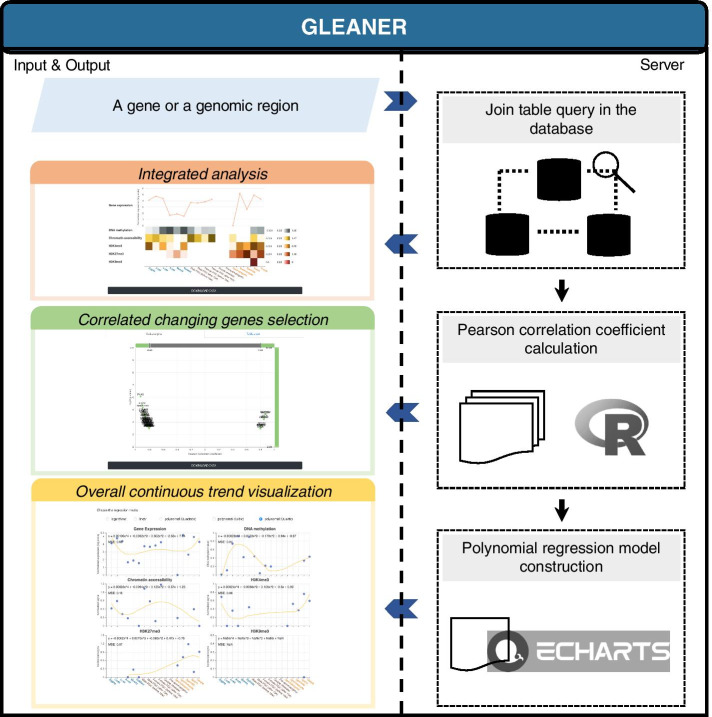
The GLEANER workflow. The workflow guides users through distinct steps of analysis in GLEANER. Users can provide a gene or a genomic region in the human or mouse genome. Once a genomic region was provided, the gene covering the region was regarded as the input gene. In the query step, the transcriptional and epigenome features of the gene in the database are selected as output. In the processing step, the PCCs between gene expression and epigenome features at each developmental phase and the whole germline cycle are calculated using R. The candidate genes with correlated changes can be selected by the users with the satisfying of the thresholds of both PCC and *p*-value. Then, the polynomial regression model construction of gene expression and each epigenome feature will be performed by the ECharts Statistics module (right). Ultimately, all integrative analysis results will be displayed in three parts, which include integrated analysis plots of transcriptional and epigenetic features, correlated gene expression selection, and visualization of continuous expression and epigenetic features (left)

The protocol of GLEANER taking a gene or a genomic region as input for integrated analysis of the transcriptional and epigenetic dynamics, selection of correlated genes, and overall continuous trend visualization during the mammalian germline cycle is presented as a workflow in Fig. 2.

#### Input

Users can begin a query by inputting a gene name in different formats (i.e., official gene symbol and RefSeq ID) or a genomic region less than 10,000 bp in the format of “chromosome:start–end”. For gene name input, GLEANER recognizes the format type for subsequent analysis. For genomic region input, the gene within the region will be used for subsequent analysis.

#### Integrated analysis

To reflect the dynamics, integrality, and continuity in the mammalian germline cycle, an integrated analysis of the input gene's expression levels, average DNA methylation levels, average chromatin accessibility signals, and histone modification features on promoters is conducted and displayed as a line plot combined with heatmaps. Separate PCCs in three phases and the overall PCCs between these features are calculated to provide clues about epigenetic regulation and interaction. This information can be downloaded by clicking the “DOWNLOAD DATA” button.

#### Correlated gene selection

Genes with similar transcriptional dynamics trends in the germline cycle may be coexpressed or share similar regulatory mechanisms. To select these correlated genes, GLEANER used the preprocessed annotation matrixes and calculated the PCCs of gene expression levels between genes. For each type of data, the PCCs and *p*-values of correlation tests are displayed as a volcano plot and a table including the significant positively and negatively correlated candidates that satisfy the adjustable thresholds of both PCC and *p*-value. To benefit the users, a button, which allows the download of the underlying data being visualized can be found at the bottom of this panel.

#### Overall continuous trend visualization

According to the continuity and cyclicity of the germline cycle, the examination of the overall continuous trends of transcriptional and epigenetic features is an effective and feasible way to investigate the transmission of genetic information between generations. Different types of regression models, which included linear model, logarithmic model, and polynomial (quadratic, cubic, and quartic) model, were built to fit the separate points across the time-series data in different stages and demonstrate the overall continuous trend of transcriptional and epigenetic features (see Methods and Materials for details). This trend was visualized as a line plot with points at distinct stages, including those cell types with unavailable data in the germline cycle. It is worth noting that signal values of ChIP-seq samples are not suitable for direct quantitative comparison due to the semi-quantitative characteristic of ChIP-seq if important parameters are unknown.

In summary, in a general workflow, GLEANER takes a gene or a genomic region as input to start a query of the transcriptional and epigenetic features in the human or mouse germline cycle. GLEANER conducts the analysis, and visualizes the results in three modules, namely, the integrated analysis of gene expression levels and epigenetic characteristics on the corresponding regulatory region, the identification of genes with correlated changes and the visualization of the overall continuous dynamic transcriptional and epigenetic trends in the germline cycle. In the cluster page, GLEANER also navigates the similarity of input genes globally by representing them in the clusters of transactional and each epigenetic feature.

### Application case 1

In this section, a tripartite transcription factor network (PRDM1, PRDM14, and AP2γ) [18] for the specification of mouse early germline cells is chosen as an example to show the functionality of GLEANER and to discuss the results in detail. It has been reported that PRDM1 and PRDM14 are mutually interdependent and induce the expression of the transcription factor AP2γ in mouse PGC development [19, 20]. This network upregulates the expression of pluripotency genes, such as *Nanog*, *Pou5f1*, and *Sox2*, leading to the initiation of epigenetic reprogramming during the specification and migration process in the mouse PGC development phase [21]. However, the epigenetic regulatory effect of this network in the PGC phase remains unclear.

**Fig. 3:**
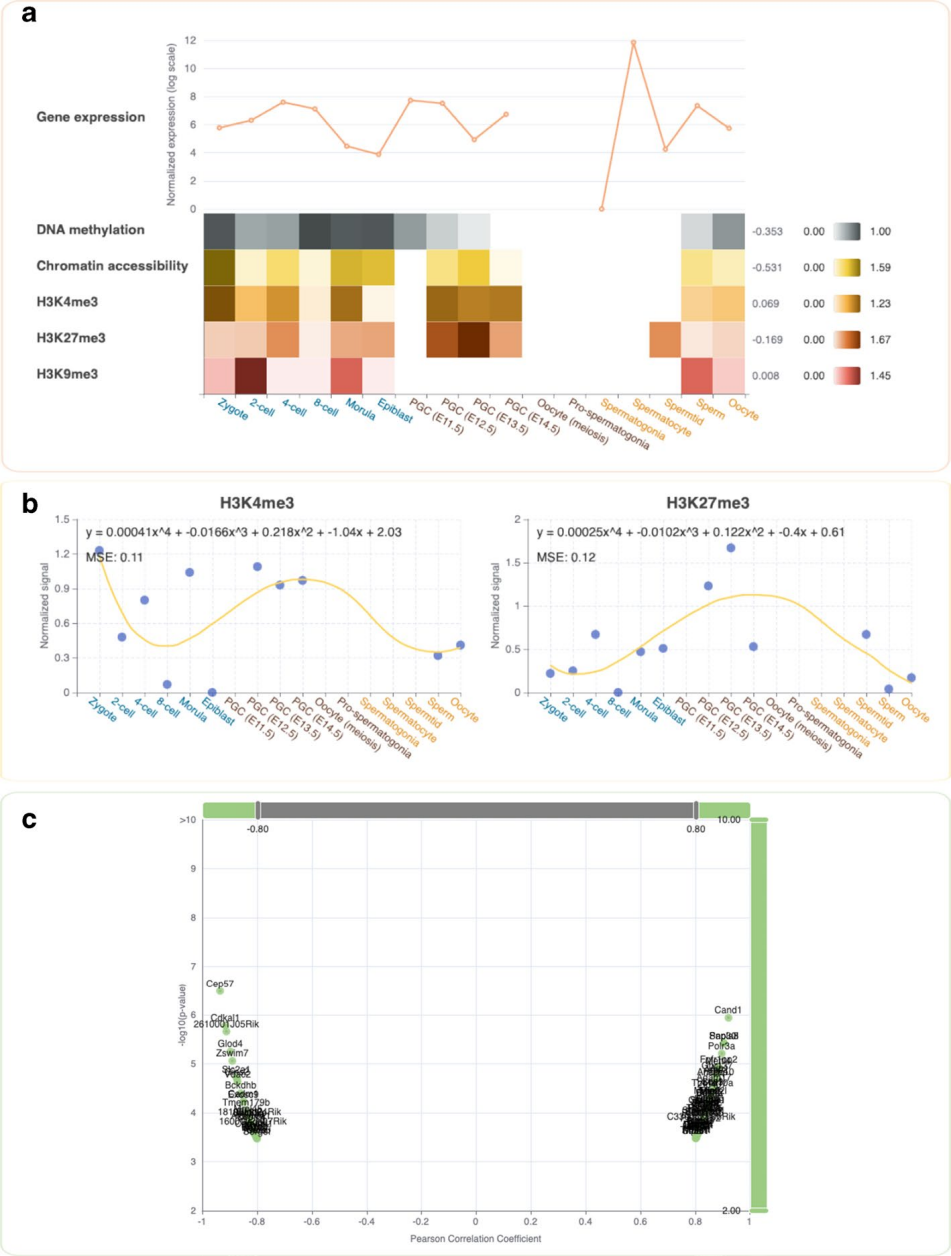
The integrated analysis and visualization of the transcriptional and epigenetic features of *Tfap2c* during the mouse germline cycle. **A** A line plot and heatmaps showing the integrated analysis of the transcriptional and epigenetic features of *Tfap2c* during mouse germline cycle. Breakpoints in the line plot indicate missing values at distinct developmental stages. Heatmaps represent the average DNA methylation levels, average chromatin accessibility signals, and histone modification features on the promoter of *Tfap2c*, while gradient color reflects the intensity of the signal, and white grids indicate missing values at distinct developmental stages. The overall PCCs between the expression levels with these features are displayed at the right of the heatmaps. **B** The overall continuous trends of the epigenetic features (H3K4me3 and H3K27me3) on the promoter of *Tfap2c*. Curves in each panel were fitted by quartic polynomial regression models. **C** A volcano plot showing the PCCs and the correlation test’s *p*-values of the transcriptional similarity between *Tfap2c* and other correlated changing genes

To investigate the potential function of epigenetic information transmission across the germline cycle phases, the coding genes of this network (*Prdm1*, *Prdm14*, and *Tfap2c*) were queried as inputs of GLEANER to explore the dynamic changes and information about potential epigenetic regulation. GLEANER first conducted an integrated analysis of the transcriptional levels and epigenetic features. In the PGC development phase, *Prdm1*, *Prdm14*, and *Tfap2c* were expressed from Embryonic Day 11.5 (E11.5) to E14.5, which was consistent with previous studies [22]. The expression trend of *Tfap2c* was positively correlated to the dynamic trend of the H3K4me3 signal of its promoter region (PCC = 0.894) (Fig. 3A) and negatively correlated to the dynamic trend of the H3K27me3 signal of its promoter region (PCC =  − 0.581). It is worth noting that the DNA methylation level on the promoter region of *Tfap2c* decreased rapidly as well as the H3K27me3 signals increased, which indicated that the dominant regulating epigenetic factors of *Tfap2c*’s expression might have changed during this period. Besides, the increasing H3K4me3 and H3K27me3 signals on the *Tfap2c* promoter from the late preimplantation embryogenesis phase to the PGC phase indicate a potential bivalent regulatory mechanism of AP2γ during these two phases of the mouse germline cycle (Fig. 3B). To further explore the other potential regulatory factors involved in this network, GLEANER selected the genes whose changes most correlated with those of *Tfap2c* at the expression level. As shown in Fig. 3C, *Cand1*, an assembly factor of SKP1-CUL1-F-box protein complexes that related to the regulation of transcription and cell differentiation processes, shows the most positively correlated expression pattern.

### Application case 2

**Fig. 4:**
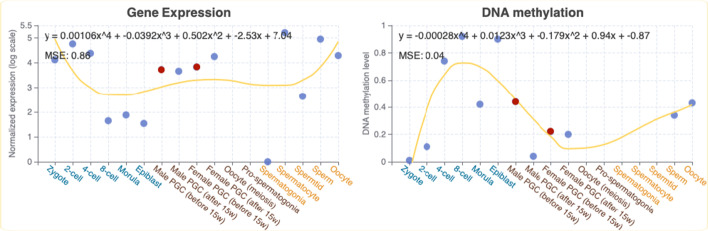
Scatter plots showing the expression level of ATRX and the average DNA methylation level on the promoter of ATRX during the human germline cycle. Curves in each panel were fitted by quartic polynomial regression models. The red dots highlight the values at the early PGC stages

In this section, an X-chromosome reactivation-associated gene (*ATRX*) at the human PGC development stage is chosen as an example to show how GLEANER provides clues about epigenetic regulatory mechanisms during the human germline cycle. It has been reported that X-chromosome reactivation is one of the representative events of the epigenetic reprogramming process during mammalian female PGC development [23–27]. However, studies also observed that the total expression level of the genes on the X chromosome in female PGCs did not reach expected twofold higher than that in male PGCs at a similar stage [28, 29], indicating that reactivation of genes in the X-chromosome of female PGCs is incomplete. A recent study also reported that the expression of X-linked genes on both alleles is transcriptionally reduced but not silenced, representing a compensated state referred to as X-chromosome dampening [30], and *ATRX* was one of these biallelically expressed X-linked genes [31]. By querying *ATRX* in the human germline cycle using GLEANER, we found that the expression level of *ATRX* was 1.07-fold higher in the early female PGCs than in the early male PGCs, which indicated that the reactivation of *ATRX* is also incomplete (Fig. 4). Besides, the *ATRX* gene promoter showed a relatively higher methylation level in the early male PGCs (average methylation level = 0.44) than in the early female PGCs (average methylation level = 0.22), suggesting that DNA methylation might be related to the incomplete or ongoing reactivation of the *ATRX* gene, which indicated that epigenetic factors were involved in X-chromosome reactivation in the human PGC development process. However, due to the lack of other epigenetic features (i.e., histone modifications), we cannot rule out the possible regulatory effects of these features on the unexpectedly low accessibility of the *ATRX* gene promoter at the early female PGC stage.

### Discussion

Although an increasing amount of multiomics data has been generated in human and mouse germline cycles, to the best of our knowledge, there is no web server to facilitate the integrated analysis and visualization of transcriptional and epigenetic characteristics in the germline cycle. In this study, we developed GLEANER, a web server for germline cycle expression analysis and epigenetics roadmap visualization. Using normalized transcriptional and epigenetic high-throughput sequencing data collected in human and mouse germline cycles, we investigated the genetic and epigenetic information that is transmitted between generations. For each input, GLEANER shows integrated information about the gene transcription, DNA methylation, chromatin accessibility, and histone modification characteristics on its corresponding regulatory region, both positively and negatively correlated changing genes during the germline cycle and the overall continuous dynamic transcriptional and epigenetic trends in the germline cycle. Case studies of a tripartite transcription factor network (PRDM1, PRDM14, and AP2γ) and an X-chromosome reactivation associated gene (*ATRX*) demonstrated the functional utility of GLEANER and highlighted that the integrative analysis of transcriptional and epigenetic features could provide a new angle to investigate the mechanisms underlying the epigenetic regulatory effects in genetic and epigenetic information transmission during the germline cycle. In addition to visualizing the characteristics of individual genes, users may also be interested in navigating relationships of multiple genes globally. In the cluster page, GLEANER performed clustering analysis for the transcriptional and each epigenetic feature, and also provided the user with the ability to query a list of genes for the clusters they belong to. By highlighting these input genes in the heatmap of the clusters, the user was given a clue to evaluate whether these genes are more inclined to be clustered, or are random.

The long time span of the germline cycle and material scarcity have largely limited the understanding of the genetic and epigenetic information transmitted during this process. GLEANER takes advantage of the continuity and integrality of the germline cycle to partially overcome this limitation through a computational approach. However, the inferred data cannot perfectly represent the actual values in the unavailable cell types in the germline cycle. With further breakthroughs in technology making more data available in the future, GLEANER will be updated to represent the cyclical change of genetic and epigenetic characteristics more completely and present the overall continuous dynamic trends more accurately. On the other hand, future data will provide more various aspects of the integrated analysis. For example, there is still little high-throughput sequencing data available from the PGC development phase (i.e., PGC migration in human and mouse early gestational stages, pro-spermatogonia, and mitotic oocyte migration in human late gestational stages). With the complement of these unavailable data, a systematic comparison of the transcriptional and epigenetic features between humans and mice can be included in future updates of GLEANER. GLEANER will be maintained and updated periodically to collect and integrate new transcriptional and epigenetic datasets in future updates.

## Conclusions

In summary, by using GLEANER, users can easily obtain integrated information on the transcriptional and epigenetic features in the germline cycle by querying a gene or a genomic region. In addition, through correlation analysis and regression analysis in GLEANER, users could not only obtain candidates with similar transcriptional and epigenetic feature dynamic changes that indicate the potential underlying mechanisms of epigenetic regulation in the mammalian germline cycle but could also predict missing features by extrapolating from the overall continuous dynamic trends of the transcriptional and epigenetic features. Thus we believe that GLEANER could be a valuable tool to help understand the transcriptional and epigenetic information transmitted in the mammalian germline cycle.

## Supplementary Information

**Additional file 1.** Framework of the GLEANER construction.

## Data Availability

All datasets analyzed during this study are included in this published article and the sources are cited accordingly. The analyzed data are available at GLEANER’s data page: http://compbio-zhanglab.org/GLEANER/data.html.

## Abbreviations

PGC: Primordial germ cell
BS-seq: Bisulfite sequencing
DNase-seq: DNase I hypersensitive sites sequencing
ChIP-seq: Chromatin immunoprecipitation sequencing
CUT&RUN: Cleavage Under Targets & Release Using Nuclease
GEO: Gene Expression Omnibus
PCC: Pearson correlation coefficient
LAMP: Linux operating system, Apache web server, MySQL database, and PHP programming language
