# Supplementary material for: GLEANER: a web server for GermLine cycle Expression ANalysis and Epigenetic Roadmap visualization
Source: BMC Bioinformatics. 2021 May 31;22:289. doi: 10.1186/s12859-021-04217-1 (PMC8165803; doi:10.1186/s12859-021-04217-1)
Supplement: Supplementary file 1 — Additional file 1. Framework of the GLEANER construction. [file 12859_2021_4217_MOESM1_ESM.docx]

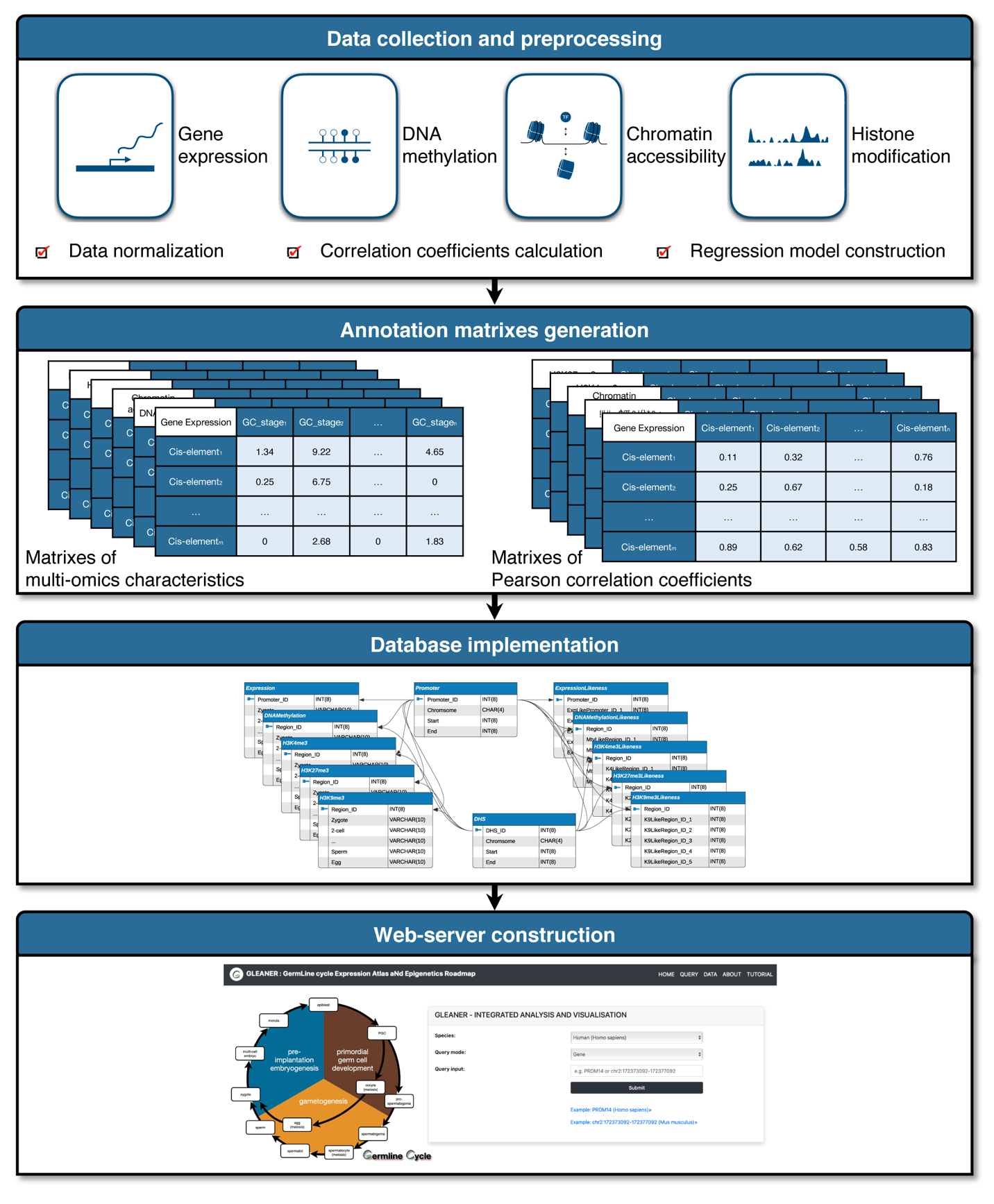


**Supplementary Figure. 1. Framework of the GLEANER construction.**

The schematic diagram represents the steps of the GLEANER construction. First, different types of transcriptome and epigenome data (gene expression, DNA methylation, chromatin accessibility, and histone modification characteristics H3K4me3, H3K27me3 and H3K9me3) were collected and preprocessed, which contained data normalization, transcriptional correlation calculation between genes and epigenetic features at each developmental phase of the germline cycle. Second, preprocessed data were annotated as standardized matrixes, including transcriptional and epigenetic feature matrixes and Pearson correlation coefficient matrixes, to facilitate downstream processing. Next, the MySQL database was established according to the annotation matrixes generated and the genetic information for table connection. Ultimately, based on the back-end fundamentals, the GLEANER web server was constructed as described above (connected with Figure 2).
